# Transposon activity, local duplications and propagation of structural variants across haplotypes drive the evolution of the Drosophila S2 cell line

**DOI:** 10.1186/s12864-022-08472-1

**Published:** 2022-04-07

**Authors:** Jacob Lewerentz, Anna-Mia Johansson, Jan Larsson, Per Stenberg

**Affiliations:** 1 Department of Molecular Biology, Umeå University, SE-901 87 Umeå, Västerbotten Sweden; 2 Department of Ecology and Environmental Sciences, Umeå University, SE-901 87 Umeå, Västerbotten Sweden

**Keywords:** Structural rearrangements, Haplotype structure, Cell-line evolution, S2-DRSC

## Abstract

**Background:**

Immortalized cell lines are widely used model systems whose genomes are often highly rearranged and polyploid. However, their genome structure is seldom deciphered and is thus not accounted for during analyses. We therefore used linked short- and long-read sequencing to perform haplotype-level reconstruction of the genome of a *Drosophila melanogaster* cell line (S2-DRSC) with a complex genome structure.

**Results:**

Using a custom implementation (that is designed to use ultra-long reads in complex genomes with nested rearrangements) to call structural variants (SVs), we found that the most common SV was repetitive sequence insertion or deletion (> 80% of SVs), with *Gypsy* retrotransposon insertions dominating. The second most common SV was local sequence duplication. SNPs and other SVs were rarer, but several large chromosomal translocations and mitochondrial genome insertions were observed. Haplotypes were highly similar at the nucleotide level but structurally very different. Insertion SVs existed at various haplotype frequencies and were unlinked on chromosomes, demonstrating that haplotypes have different structures and suggesting the existence of a mechanism that allows SVs to propagate across haplotypes. Finally, using public short-read data, we found that transposable element insertions and local duplications are common in other *D. melanogaster* cell lines.

**Conclusions:**

The S2-DRSC cell line evolved through retrotransposon activity and vast local sequence duplications, that we hypothesize were the products of DNA re-replication events. Additionally, mutations can propagate across haplotypes (possibly explained by mitotic recombination), which enables fine-tuning of mutational impact and prevents accumulation of deleterious events, an inherent problem of clonal reproduction. We conclude that traditional linear homozygous genome representation conceals the complexity when dealing with rearranged and heterozygous clonal cells.

**Supplementary Information:**

The online version contains supplementary material available at 10.1186/s12864-022-08472-1.

## Background

*Drosophila melanogaster* is a powerful model system in which, as in other organisms including humans, cell lines serve as important research tools [1, 2]. Several widely used cell lines have been established within the genus *Drosophila* [3]. However, like human cancers, cell lines evolve non-uniformly and display great heterogeneity [4], as demonstrated by their variety in genome ploidy and copy number [5], transcriptional diversity [6], and propagation of transposable elements [7]. Because cell lines are often used in research as a proxy for the organism, it is important to understand their genomic evolution to ensure appropriate experimental design and analysis. However, most published cell-line research on *Drosophila* has used the progenitor genome as a reference without taking cell-line specific rearrangements into consideration.

Until recently, whole-genome DNA sequencing has primarily used short-read technologies (e.g., Illumina) that enable accurate identification of some types of mutations including single- and oligo-nucleotide polymorphisms, and copy-number variants. However, due to their short read lengths, large structural variants (mutations > 50 bp, SVs) cannot be resolved and are difficult to link when using such technologies. Consequently, they generally cannot provide a global overview of genomic rearrangements or haplotype structure. In addition, to understand the evolution and genomic structure of highly rearranged polyploid cell lines, it is essential to identify mutations with haplotype resolution. The recent introduction of technologies that sequence long DNA fragments (Pacific Biosciences, Oxford Nanopore Technologies, and barcoded Illumina-based sequencing; 10X Genomics) has revolutionized the field of genomics and enabled the unprecedented identification of large SVs [8–11]. The long fragment lengths of these technologies also make it theoretically possible to link SVs [12] and SNPs [13] to reveal haplotype structure. However, there is currently a lack of software capable of analyzing long reads from polyploid and highly rearranged genomes [11].

Cancers must overcome several hurdles to become immortalized. The path to immortality varies but commonly involves evading apoptosis while acquiring self-sufficiency in growth signals, insensitivity to anti-growth signals, limitless replicative potential, and population-level functions that sustain angiogenesis and enable tissue invasion and metastasis [14]. Less is known about the commonly used *Drosophila* cell lines, but they have several common features including onset of pathways involved in growth and survivability, offset of differentiation pathways [6], and gain of anti-apoptotic functions and cell-cycle regulators [5]. Even less is known about the mechanisms that create the functional and genetic diversity needed for selection early in the evolution of cell lines. However, mobilization of transposable elements [7] and duplications of genetic material leading to increases in copy number [5] are both frequently observed. Better data and computational tools are needed to fully characterize cell-line genomes at the haplotype level and to thereby start unravelling the molecular mechanisms driving these rearrangements.

In the *Drosophila* cell-line community, Schneider’s Line 2 (S2) and its variants represent the most used. This cell line was derived from a male but has since lost the Y chromosome [5], has become tetraploid, gained a vast number (> 30%) of copy number aberrations, and has an elevated number of transposable elements [15]. The S2 cell line originates from embryonal tissue and has two siblings (S1 and S3) that were derived from the same fly stock. Within the S-cell lineage, a multitude of stocks exist that diverge (including S2R+, a clonally derived expansion of S2) by copy number [5] and transposable elements [7]. Although S2 cells are widely used in research, a comprehensive analysis of rearrangements, haplotype structure, and the mechanisms that enabled the cell line to acquire its highly rearranged state is still lacking.

Here, we study the genome structure of *Drosophila melanogaster* cell line S2-DRSC using short- and long-read sequencing technologies. While transposable element abundance and copy number has been assessed in S2, a comprehensive study of rearrangements and haplotype structure does not exist. Here, the relative abundance of SV types is determined: transposable elements dominate, followed by sequence duplications, and very few translocations. Since transposable elements and copy-number variation are abundant in many cell lines, we explored the relation between multiple *Drosophila* cell lines based on the prevalence and location of these metrics together with SNPs and local duplication signatures using public short-read data. Expanding on previous knowledge of transposable elements, our data shows that the insertion loci of these elements are distributed across the genome. Gain of copy-number is preferentially acquired through local (as opposed to distant insertion) sequence duplication. The haplotypes are homozygous at SNP level but heterozygous by structural variants. To infer the rearrangement mechanisms of S2-DRSC, we hypothesize that local sequence duplication has originated through re-initiation of replication and that mitotic recombination (based on varying haplotype frequency of variants that emerged during the cell-line’s evolution) has enabled the cell line to propagate variants across haplotypes. Mitotic recombination would enable a cell line to unlink mutations and allow advantageous mutations to propagate to additional haplotypes independently of neighboring deleterious mutations.

## Results

We cultured S2-DRSC cells and performed DNA sequencing using multiple technologies (See Table 1 for sequencing statistics). By HiSeq-X Illumina sequencing of a DNA library prepared according to the 10X Genomics Chromium protocol we obtained linked (barcoded) short-reads. By Pacific Biosciences RSII (Pacbio) and Oxford Nanopore Technologies (ONT, using both standard and custom ultra-long read protocols, see Table 1 for a comparison) we obtained long-reads. To confirm our cell-line stock, we first calculated copy-number variation using linked short-reads data and compared the variation to those previously published [5] datasets of S2-DRSC and other cell lines (Additional file 1: Fig. S1). We conclude that our S2-DRSC stock agrees with previously published datasets of S2-DRSC and that, although these three datasets relate to supposedly identical S2-DRSC cells, they are only 74% identical and therefore have a copy-number discrepancy of 26%.

**Table 1:** Summary of sequencing datasets

Dataset	N50	N50 (> 1 kbp)	Coverage	Coverage (> 1 kbp)	Coverage (> 10 kbp)	Coverage (> 100 kbp)	Bases (Billions)	Sequences (Millions)	% > 100 kbp of > 10 kbp
Linked reads	NA	NA	193.6	NA	NA	NA	29.05	104 × 2	NA
Pacbio	16.99	17.001	114.2	114.0	90.3	NA	17.13	1.51	NA
Nanopore	21.22	22.089	24.2	23.6	16.3	0.19	3.63	0.57	1.1
Nanopore (long)	23.68	24.403	153.5	150.3	117.0	12.0	23.02	2.91	10.3

### Chromosomes harbor wide-spread LTR retrotransposon insertions and local sequence duplications

To call SVs from long-reads, we used a custom logic (that is read-centered and use the full information of ultra-long reads, briefly outlined in Additional file 1: Fig. S2A and described in the Methods) that resulted in 41,463 SV calls, of which 5376 remained after filtering for low-frequency events. We first classified SV calls by the length of the inserted sequences (Additional file 1: Fig. S2B). The majority (79.4%, *N* = 4268) of the calls corresponded to insertion of a > 1 kbp sequence, most of which (86.9%, *N* = 3710) were 1–10 kbp long. Insertions > 1 kbp were classified in terms of overlap with annotated repetitive sequences (Additional file 1: Fig. S2C), revealing that most of them (95.6%, *N* = 4081) contained 75–100% repetitive sequences. To determine the distance between rearrangement breakpoints in the reference genome, calls were divided into six classes; the first class contained trans-chromosomal rearrangements and the remaining five corresponded to intra-chromosomal distances of different magnitudes (Additional file 1: Fig. S2D). Most (94.2%, *N* = 5063) of the rearrangements had breakpoints < 10 kbp apart; only a few (0.2%, *N* = 11) were trans-chromosomal. We next classified all SVs based on the type of event they represented: repetitive deletions or insertions > 1 kbp (82.8%), local duplications (7.7%), short insertions or deletions < 1 kbp (3.2%), or unclassified (6.3%). Unclassified events are all events that our read-centered approach cannot determine (or events nested inside other events), such as large duplications, deletions of non-repetitive sequence, and inversions. However, most of these events seem to be large local duplications (see below). Finally, we calculated the number of SV breakpoints per Mbp and chromosome (Additional file 1: Fig. S2E) to determine the density of rearrangements on chromosomes, revealing that all chromosomes had > 30 SVs/Mbp (about 1 SV per 33 kbp). To conclude, all chromosomes have many rearrangements, most of which are related to repetitive sequences and are local (breakpoints < 1 kbp). Since most SVs are associated with repetitive sequences and duplication events, we focus on these in the following analysis.

**Fig. 1:**
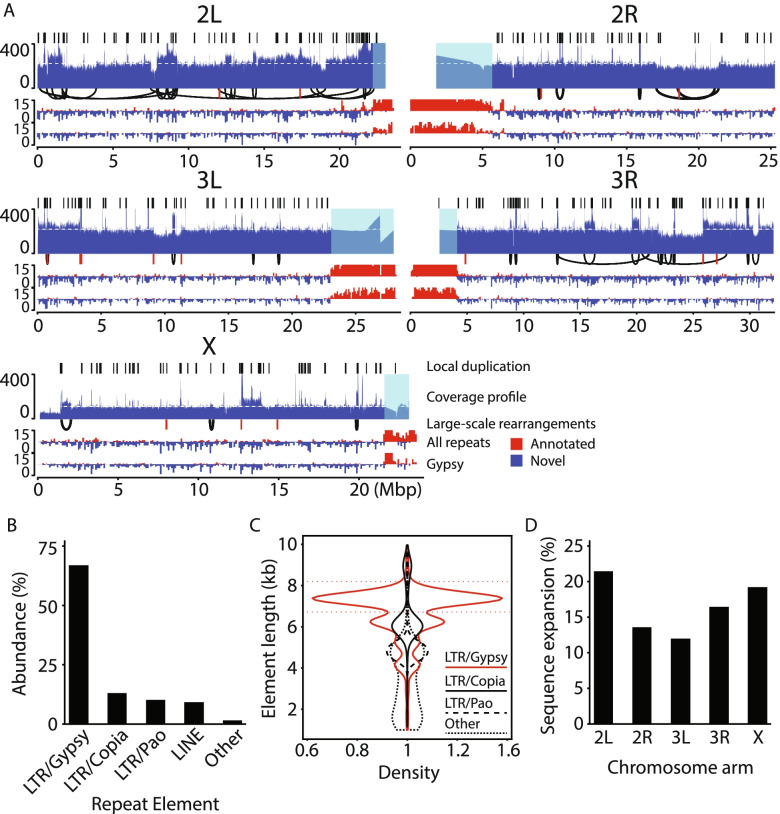
Chromosomal overview of rearrangements called by long-reads and novel transposable element insertions. **A** Chromosomal overviews showing local duplication calls, read coverage (white line shows autosome or sex chromosome median and shadings toward the centromere denote repetitive regions in which read coverage was not used to compile the coverage profile), large-scale (> 100 kbp breakpoint distance) rearrangements (deletions, inversions, duplications; shown in black, and translocations; shown in red), and repetitive sequence density in 100 kbp bins (red blocks above the line denote reference genome annotations; blue blocks below the line denote novel insertions) of all repeat annotations and *Gypsy* elements. Unresolved large-scale rearrangements (not spanned by a read) can be interpreted using the coverage profile. Increased coverage indicates duplication while decreased coverage indicates deletion. Regions annotated as repetitive (e.g., centromere- and telomere-proximal regions) are not analyzed. **B** The abundance (Y-axis) of insertions > 1 kbp in length with > 90% classification to a single repetitive element (X-axis). Only the four most abundant element classes are shown individually; the rest are pooled into “Other”. **C** Violin plot showing the insertion length (Y-axis) distribution density (X-axis) per element as individual violins. Thin dashed red lines show insertions comprising 90–110% of a full-length *Gypsy* element. **D** Chromosome sequence expansion in percent due to *Gypsy* insertions

To visualize the chromosome-level genomic landscape, we computed tracks showing local sequence duplication, coverage, large rearrangements (> 100 kbp distance between breakpoints in the reference genome, including those that are unclassified), and repetitive insertion density together with the density of repetitive sequences annotated in the reference genome. This revealed that local duplications and repetitive insertions were distributed across the chromosomes (Fig. 1A). However, the logic implementation underestimates the prevalence of local duplications since it requires at least one read to span the event (a duplicated region of 100 kbp would require a read > 200 kbp in length to contain both duplicates plus anchoring sequence on both sides of the duplication). This is reflected in the increased regional read coverage at sites with unclassified SV calls larger than 100 kbp (examples include the 12.5 Mbp region of chromosome arm 2L, the regions at 10-, 14- and 16 Mbp on 2R, the region at 10.5 Mbp on 3L, the regions at 16- and 20 Mbp on 2R, and the region at the telomere tip of X). We conclude that repetitive sequence insertions and local sequence duplications are widespread in the genome and represent the most common type of SVs. Gain of sequence via local duplication appears to be the main mechanism driving copy-number gain.

The high prevalence of repetitive insertions motivated us to identify the repeat elements. To this end, we focused on insertions > 1 kbp (79.4% of all SVs, *N* = 4268) of which > 90% was annotated to one repeat element (69% of all SVs, *N* = 3707). We found that the *Gypsy* long terminal repeat (LTR) retrotransposon element was the most abundant (67.1%, *N* = 2488) repeat class in this group (Fig. 1B). The total number of insertions classified to the LTR family (*Gypsy*, *Copia*, and *Pao*) was 3324 (61.8% of all SVs). The distribution of *Gypsy* element insertion loci is shown in Fig. 1A. Notably, we observe a high (49.9%) prevalence of full-length *Gypsy* elements (having 90–110% of the annotated *Gypsy* element length [16]) not annotated in the reference genome (Fig. 1C) together with a drastic chromosomal sequence expansion (e.g., > 20% on chromosome arm 2L) due to *Gypsy* elements (Fig. 1D). These findings indicate that a rapid burst of *Gypsy* activity occurred during the cell-line’s evolution.

We next asked how copy-number gains and disrupting rearrangements re-shaped gene functions in S2-DRSC. Copy number in 50 bp genomic bins was computed using alignments from long-read datasets. Genes were classified either as gained (copy number greater than chromosome ploidy) or disrupted (breakpoints disrupting coding sequence present in all haplotypes). Classified genes were functionally clustered using DAVID [17] and visualized using REVIGO [18]. Gained gene functions (Additional file 1: Fig. S3A) included “Nucleosome assembly”, “Regulation of protein serine/threonine phosphatase activity”, “DNA-templated transcription, initiation”, “Body morphogenesis”, and “Lysosomal transport”. Disrupted gene functions (Additional file 1: Fig. S3B) included “Cell wall macromolecule catabolism”, “Defense response”, “Potassium ion transport”, “Sensory perception of smell”, “Morphogenesis of a polarized epithelium”, “Establishment or maintenance of cell apical/basal polarity”, and “Cytolysis”. For GO-term *p*-values, see Additional file 2. We speculate that the cell-line’s expression patterns were tuned to upregulate genes related to genome organization and rapid cell cycle and that disruption appears to be tolerated in tissue-specific and developmental genes.

**Fig. 2:**
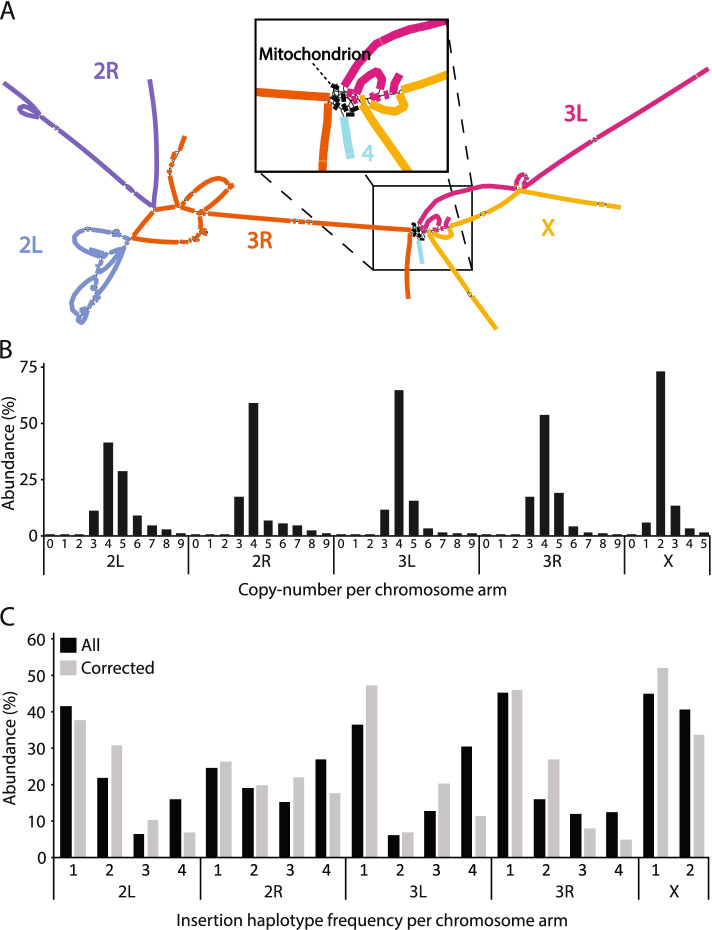
Genome graph, copy-number and haplotype frequency of insertions. **A** Graph representation of the genome generated using large-scale (> 100 kbp breakpoint distance or translocation) SVs. Segments are colored according to alignments to the reference genome chromosome. Connections between segments are established from large-scale SVs and are indicated with black lines. Haplotype structure can be reconstructed by graph traversal, where forks in the graph reflect different haplotypes. **B** Distribution in percent (Y-axis) of copy number in 50 bp genomic bins per chromosome (X-axis). **C** The chromosomal haplotype frequency (X-axis) and the abundance in percent (Y-axis) of inserted repetitive sequences (breakpoints < 1 kbp) in tetraploid regions. Rearrangement haplotype frequency were determined by counting read support. Black bars represent the distribution of all insertions and gray bars represent the distribution of insertions absent in the source fly stock (based on short-read analysis of insertions in S1, S2-DRSC, and S3)

To produce a visual overview of the genome structure, a graph representation was generated based on large (> 100 kbp breakpoint distance) SVs (Fig. 2A). The genome graph cannot be resolved using a single path, demonstrating that significant variation between haplotypes exists at larger scales. This structural variation includes large rearrangements within and between chromosomes. Interestingly, the graph also revealed mitochondrial sequence insertions (Fig. 2A, zoom-in; See Additional file 1: Fig. S4 for more details). Trans-chromosomal rearrangements were always associated with copy-number gain (Additional file 1: Fig. S5). We thus conclude that using the linear *D. melanogaster* reference assembly when analyzing cell lines such as S2-DRSC may lead to incorrect conclusions.

### Haplotypes are homozygous by SNPs but heterozygous by SVs and allow the propagation of SVs

To reconstruct the haplotypes of the genome, a de novo assembly is preferred. However, it was not possible to produce a contiguous assembly using long-read data. To roughly estimate the impact of a heterozygous haplotype structure on assembly fragmentation, we generated mock datasets of pooled fly long-read datasets from publicly available data [19] (Additional file 3) and used them to perform de novo genome assembly. Despite pooling multiple fly datasets, we obtained contiguous de novo assemblies, although they were more fragmented than individual dataset assemblies (Additional file 1: Fig. S6, see Additional file 4 for assembly statistics). However, an assembly obtained from a pooled dataset representing four different species (*D. simulans, D. sechellia, D. yakuba,* and *D. erecta*) separated by up to 10 million years [20] exhibited similar fragmentation to the cell line. While not a perfect comparison, this indicates that the haplotype structure of S2-DRSC is complex. Since assemblers rely on heterozygosity between haplotypes (and that this signal is stronger than the inherent per-base error rate of long-reads) to separate them as individual contigs, we next investigated the variation between haplotypes at the single nucleotide polymorphism (SNP) level using linked short-reads data. We selected SNPs in tetraploid regions (determined by coverage) and excluded SNPs supported by all reads since these were most likely present in the progenitor fly. Remarkably, we found relatively few SNPs in one, two, or three haplotypes (read support percentage of 12.5–37.5%, 37.5–67.5% or 67.5–87.5%, respectively); most SNPs were present in < 1 or > 3 haplotypes (Additional file 1: Fig. S7). The most likely explanation for the predominance of < 1 and > 3 haplotype SNPs is that these reflect the presence of different clones in the culture. Increasing the read support threshold for SNP calling sharply reduced the number of SNPs with < 1 or > 3 haplotypes (see Additional file 1: Fig. S8). This indicates that clones containing sequence variants deviating from the major clone genotype exist in very low frequencies. We conclude that the cell population has a dominant clone with largely homozygous haplotypes at the SNP level and therefore make no further attempts to assemble the genome.

We next investigated the zygosity of the cell line, motivated by the inconsistency between the homozygosity suggested by the SNP data, the heterozygosity suggested by the genome graph (Fig. 2A) and not being able to reconstruct the genome via de novo assembly (indicative of heterozygous haplotype structure). To investigate the zygosity of sequence duplications, we visualized the distribution of copy number per chromosome (in 50 bp genomic bins, from long-read data, shown in Fig. 2B). Figure 2B shows that all chromosomes primarily display the expected copy number: four for tetraploid autosomes and two for the diploid X chromosome. Copy-number gains and losses mainly occurred in steps of one and thus represent events on single haplotypes (Fig. 2B, see also Additional file 1: Fig. S9). The substantial (82.7%) prevalence of single (one) copy-number alterations from base ploidy suggests that sequence duplications are heterozygous and that the haplotypes have a unique structure.

We reasoned transposable elements could be queried for zygosity since these can be preferentially selected in regions without a simultaneous duplication or deletion (that change copy number and thus confound the separation of a regional copy vs. haplotype). To determine the zygosity, local (breakpoints < 1 kbp apart) insertions in tetraploid regions were targeted. Regions that underwent multiple copy-number altering rearrangements (or were distant inserted copies) may still have been included but are expected to be rare compared to regions of true tetraploidy since rearrangements other than local duplication and transposable element insertion were rare (< 10%, see above). Regions were classified as tetraploid based on long-read coverage (87.5–112.5% of tetraploid coverage) in 3 kbp windows over the insertion breakpoints. Insertion haplotype frequency were inferred by counting read support. Insertions were grouped by haplotype frequency and their abundance was visualized in a bar plot (Fig. 2C). We found that insertions occurred at all haplotype frequencies (one to four), indicating heterozygosity. Note that insertions in all haplotypes cannot be assumed to have occurred during cell-line evolution. We cannot directly investigate the progenitor fly, but we can identify mutations likely to have occurred during the evolution of S2-DRSC by comparison to two other cell lines (S1 and S3) established from the same genotype [21]. Transposable element insertion calls were obtained from short-reads of S1, S2-DRSC, and S3 using TEFLoN [22]. Insertions identified by long-reads were matched against short-read calls within 250 bp and required to (1) be validated with S2-DRSC short-read calls, (2) not be called in S1 or S3, and (3) have reads spanning the insertion locus in S1 or S3 (Fig. 2C, gray bars). Of 1746 insertion calls, 684 were cross-validated (point 1) and 424 were specific to the S2-DRSC cell-line (points 2 and 3). The discrepancy between insertion calls from long- and short-reads did not appear biased by the haplotype frequency (Additional file 1: Fig. S10). This analysis shows that insertions that occurred during cell-line evolution exist in all haplotype frequencies. Although possible, it is very unlikely that independent insertions would occur at the same position in multiple haplotypes. Therefore, since the haplotype frequency insertions ranges between one and four on all autosomes (one and two on X), we hypothesize that mitotic recombination is the most likely mechanism by which the haplotype frequency of rearrangements increased or decreased. Mitotic recombination in cell lines and cancers would allow them to escape Muller’s Ratchet, i.e., the accumulation of deleterious mutations in asexual populations [23]. We conclude that chromosomes have both hetero- and homozygous regions whose co-occurrence is best explained by mitotic recombination.

### Cell line evolution

Above, we showed that most rearrangements in S2-DRSC are either transposon insertions or copy-number gains via local duplication. This led us to ask whether such rearrangements also dominate in other *D. melanogaster* cell lines. We therefore downloaded public short-read paired-end datasets for multiple *D. melanogaster* cell lines (Additional file 3). For some cell lines, multiple datasets were available and downloaded. Our linked short-read dataset was used without barcodes as a standard short-read dataset, and we also included a fly dataset (Oregon R) to the cell-line comparison. We called transposon insertion using TEFLoN. Because no public long-read data existed for other *D. melanogaster* cell lines, we developed a custom script to call signatures of local duplication using paired-end short-reads (for details, see Materials and Methods). Copy-number calls were obtained from published data [5]. We defined a copy-number gain as ≥150% of the base chromosome arm copy number, in other words we measure segments that vary in copy number relative to their chromosome. The abundances of each event type after subsampling into two dataset groups to an equal read depth (one at around 12X coverage and another at around 4X coverage, see Additional file 5 for details) are shown in Additional file 1: Fig. S11, where a gray shading marks the datasets of low coverage. We note that the subsampling is based on coverage, meaning that datasets with longer reads will have fewer pairs. Since read pairs are used to call transposon insertions and local duplications, the call abundances cannot directly be compared between datasets that differ in read length and subsampling depth. Still, this analysis indicates that local duplication has occurred in cell lines other than S2-DRSC. Cell lines also varied in their abundance of copy-number gains. Overall, these results suggest that *D. melanogaster* cell lines evolved by different mechanisms. For example, S1 cells showed the greatest copy-number gain but had only a moderate abundance of local duplications, indicating that sequence gains in this line occurred via some other mechanism(s), while S2 and S2R+ were more likely to have gained sequences via local duplication than other cell lines.

**Fig. 3:**
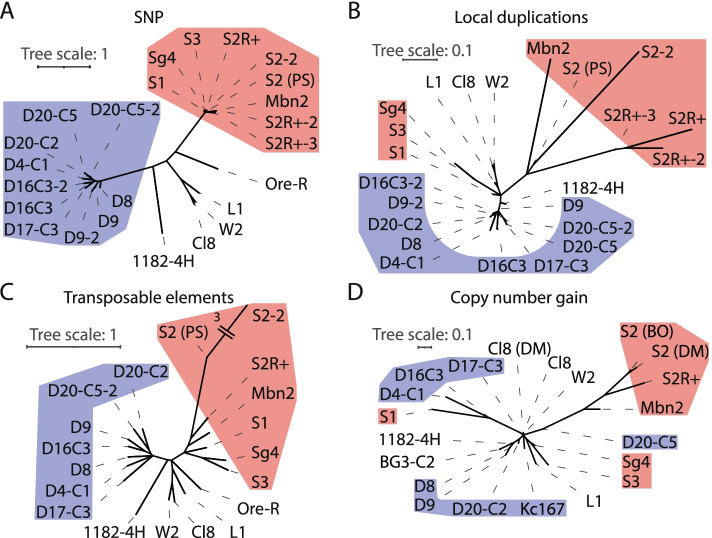
Comparison of cell line evolution. Phylogenetic trees reconstructed from binary matrices (presence or absence in 1 kbp genomic bins) of **A** SNPs, **B** local duplications, **C** transposable element insertions, and **D** copy-number [5] gains of cell lines. Branches with less than 70% bootstrap support are collapsed. The tree scale is indicated above each tree. The sequence dataset presented here is indicated by PS. In **A-D**, S-related cell lines are highlighted in red and blue D-related cell lines in blue

To see if these differences reflect different underlying evolutionary strategies, we compared the cell-line samples using a phylogenetic approach based on rearrangement types (transposon insertion, local duplication, and copy-number gain). To determine the expected relationship between cell lines, we called SNPs from the datasets and reconstructed a phylogeny (Fig. 3A). We used this strategy because documentation of cell-line history can be poor. The phylogenetic tree reflects differences between the source fly stocks used to generate the various cell lines. Only within cell lines from the same source stock can we infer the cell-line history (e.g., the S- and D-lines in Fig. 3A, which are marked in red and blue, respectively). The S-lines (S1, S2-DRSC, and S3) were established from *D. melanogaster* embryos of the same stock [21], and variants derived from S2 were clonally expanded into new stocks. The S2R+ cell line is reported to be a derivative of S2 [24]. These samples grouped as expected (Fig. 3A, see Additional file 1: Fig. S12 for a zoom-in on S-lines).

Having established the relationships between the cell lines, we studied their similarities with respect to transposon insertion, local duplication, and copy-number gain. Cell lines generally clustered in accordance with the SNP-based phylogeny (Fig. 3A) based on both local duplications (Fig. 3B) and transposon insertions (Fig. 3C). The largest differences in cell-line clustering were observed in copy-number gain (Fig. 3D). Thus, only copy-number gain yielded a cell-line clustering differing markedly from the SNP-based phylogeny. The clustering of cell lines from different sources indicated that these cell lines adopted similar routes to autonomy; for example, the S1, D4, and D16 lines were grouped, as were the S3, Sg4, and L1 lines. The branch lengths between multiple novel cell-line clusters exceed those expected by random similarity, indicating that multiple routes to autonomy exist.

## Discussion

In this work we characterized mutations in *D. melanogaster* cell line S2-DRSC with haplotype resolution using Nanopore and Pacbio long-read sequencing, and 10X Genomics long-range barcoded Illumina sequencing. We show that the haplotypes are homozygous at the nucleotide level (< 50 bp) but heterozygous on larger scales (> 500 bp). Furthermore, we propose three evolutionary mechanisms to explain the observed haplotype similarities and differences; transposon activity, gain of sequence via local duplication, and mitotic recombination events. The latter enabled the S2-DRSC line to avoid accumulating deleterious mutations and increase the haplotype frequency of advantageous mutations.

The S2-DRSC genome is highly rearranged and complex, with nested rearrangements. The number of *Gypsy* elements in the genome has increased dramatically (recently shown in [7] based on short-reads). The lengths of inserted LTR elements are preferentially close to the full element length for elements of *Gypsy* and *Pao* but not *Copia* (Fig. 1C; 55.8 and 15.5% within 90–110% of full length for *Pao* (8.8 kbp) and *Copia* (5.2 kbp)*,* respectively) which suggests that insertions classified as *Copia* are mostly inactive elements. If recombination between repetitive elements were the major mechanism driving the evolution of S2-DRSC, we would not expect a strong bias towards sequence duplication unless there was a strong negative selection for deletions. The same would be true if most events resulted from the repair of double-stranded DNA breaks induced by transposition. Interestingly, many features of the S2-DRSC genome are also seen after the re-initiation of replication origins [25–28]: (1) the genome is mainly duplicated and not deleted, (2) rearrangements predominantly maintain strandedness (Additional file 1: Fig. S13), (3) rearrangements are preferentially local rather than distant, and (4) distant rearrangements occur preferentially in duplicated regions [27]. In addition, transposon activity in maize was linked to induction of re-replication leading to local duplications [28]. Based on these observations, we speculate that most of the rearrangements found in the S2-DRSC cell line were caused by multiple re-replication events, generating random local duplications and subsequently larger rearrangements in duplicated regions, possibly facilitated by *Gypsy* transposition. This does not exclude the (likely) possibility that some of the observed rearrangements are due to other mechanisms such as recombination events between repetitive sequences.

It is assumed that clonally reproducing cells will quickly accumulate deleterious mutations that cannot be unlinked from rare advantageous mutations. Whole genome duplications can provide some resilience to this process [29, 30], but cell lines should eventually succumb to the so-called Muller’s ratchet [23]. However, our results show that individual mutations can propagate across haplotypes. We argue that mitotic recombination is the mechanism most likely to be responsible for this, and that it may provide opportunities to eliminate disruptive mutations while propagating favorable ones.

To compare the S2-DRSC cell line to other *D. melanogaster* cell lines, we identified SNPs, transposable element insertions, local duplication signatures, and copy-number gains in available short-read datasets (see Additional files 3 and 5). Our analysis of these SVs corroborated the documented history of all S-lines except for Sg4 and Mbn2. A mislabeling of Sg4 and Mbn2 cell-lines were recently reported in [7] and in line with our findings; these more likely originate from S3 and S2, respectively. Our analysis also shows that all of the studied cell lines had undergone transposable element insertions (reported by [7] in more detail), and most showed evidence of local duplications and copy-number gain, suggesting that these are universal events during cell-line evolution. Uniquely, the clustering of cell lines based on copy-number gains (Fig. 3D) differed from that based on their evolutionary history (Fig. 3A). This indicates that cell lines of different origin can follow similar evolutionary paths. Selecting an evolutionary path must induce selection on a wide range of loci for this signal to overcome the large number of random copy-number gains likely to have occurred. Since analysis of local duplications (Fig. 3B) was inconsistent with that of copy-number gains (Fig. 3D), it remains to be seen whether other events generated copy-number gains or whether the available short-read data are insufficient to identify local duplications. Only local duplications between 3 and 50 kbp were investigated, and transposable element insertions in duplication breakpoints make such duplications undetectable using short-read data. We stress that the limitations of short-reads for calling transposon insertions and local duplications hinders downstream analysis. Because long-read datasets do not exist for other *D. melanogaster* cell lines, a proper comparison of cell lines is currently not possible.

Since cell lines are common model systems, we expect that more long-read datasets will be produced to fully characterize their genomes. Using a custom algorithm and long-read sequencing data, we have shown that the S2-DRSC cell line (and likely other cell lines) is highly rearranged and has a complex haplotype structure. Our logic implementation is tailored to analyze repetitive and regionally duplicated polyploid genomes (or pooled samples) with a high density of rearrangement breakpoints, and thus complements existing software that classifies more SV types. The logic differs from existing software in two main ways. First, all-vs-all read mappings are used to identify which reads to use in SV calling and to count read support. Second, adjacent read alignments are clustered before SV calling (rather than relying on pairwise traversal of alignments). We found that this “read-perspective” approach generated non-redundant calls while minimizing the occurrence of false calls due to short alignments. To obtain valid results when using cell lines as model systems, it is essential to account for their genomic rearrangements because analysis of sequencing-based data mapped to an incorrect reference genome could lead to false interpretations. Thus, in addition to better datasets, there is a need for new software that can properly identify rearrangements and correctly visualize genome structure. Because many cell lines are polyploid and structurally heterozygous, a graph representation of the genome is likely to be more appropriate than a linear homozygous representation. The impact of large-scale rearrangements is being increasingly recognized but is likely underestimated due to the limitations of current analytical software and datasets. Consequently, our understanding of these rearrangements at the haplotype level is lacking. Since cell lines (and cancers) are commonly both polyploid and rearranged, it is important that both haplotype structure and large-scale rearrangements gain more attention.

## Conclusions

The S2-DRSC cell line genome evolved through a mobilization of *Gypsy* LTR transposons and local sequence duplications (that we speculate resulted from a DNA re-replication process) followed by large-scale rearrangements. Remarkably, very few SNPs could have had an important role in the evolutionary process and we hypothesize that the impact of mutations was probably fine-tuned by mitotic recombination and selection.

Cell lines are convenient model systems for which we need accurate reference genomes to map data. Our study shows that short-reads and commonly-used algorithms are insufficient to reconstruct cell-line specific reference genomes. However, even when using ultra-long reads and appropriate algorithms, the way cell lines are analyzed needs to be reconsidered; a linear homozygous reference will rarely provide an adequate representation of a cell-line’s genome and alternative approaches are needed.

## Methods

### DNA preparation

DNA for PacBio and 10X Genomics was isolated using the Genomic Tip 100/G Kit (QIAGEN), following the protocol for Cultured Cells in the QIAGEN® Genomic DNA Handbook. A frozen stock of Schneider’s *Drosophila* line 2 cells (S2-DRSC) was obtained from the *Drosophila* Genomics Resource Center (stock #181). We cultured the S2-DRSC cells at 25 °C in Schneider *Drosophila* medium modified with L-glutamine (LONZA), supplemented with 100 U/ml Penicillin G and 100 μg/ml Streptomycin sulfate. We initiated the protocol with 2 × 10^7^ S2 DRSC cells and the DNA was finally dissolved in 200 μl TE (10 mM Tris-HCl pH 8.0, 1 mM EDTA).

For Nanopore sequencing we wanted to extract longer DNA molecules. We therefore modified the protocol for long-read sequencing on MinION (developed by Josh Quick) for the Nanopore WGS Consortium [31]. S2-DRSC cells were grown under the same conditions as above and using media supplemented with 10% Heat-inactivated Fetal Bovine Serum. 0.5 × 10^9^ cells were washed in PBS (137 mM NaCl, 2.7 mM KCl, 10 mM Na_2_HPO_4_, 1.8 mM KH_2_PO_4_) and pelleted for 10 min at 1500×g. The cell pellet was snap-frozen in liquid nitrogen and stored at − 80 °C. We then resuspended the cells in 100 μl sterile PBS and added 10 μl TLB (100 mM NaCl, 10 mM Tris-HCl pH 8.0, 25 mM EDTA pH 8.0, 0.5% (w/v) SDS, 20 μg/ml RNase A (Qiagen)). The cells were mixed by vortexing at full speed for 5 s and incubated at 37 °C for 1 h to lyse the cells. Proteinase K (20 mg/ml) was added to a final concentration of 100 μg/ml and mixed by flipping the tube 10 times, followed by 3 h incubation at 50 °C during which the suspension was mixed every hour.

Fifty milliliter MaXtract High Density tubes (QIAGEN) were centrifuged for 2 min at 1500×g in room temperature. The 10 ml cell lysate together with 10 ml Phenol/chloroform was added to the MaXtract column and mixed into a homogenous solution, then the phases were separated at 1500×g for 5 min. The upper H_2_O-phase containing the DNA was transferred to a new MaXtract tube, mixed with 10 ml Phenol/chloroform, and separated as described above. The DNA was precipitated by adding 4 ml ammonium acetate (5 M) and 30 ml of ice cold EtOH (100%) to the DNA-phase.

The DNA was collected with a glass hook, submerged in 70% EtOH, and transferred to an Eppendorf tube. We washed the DNA with additional 1 ml 70% ethanol and then spun it at 10,000×g to remove as much EtOH as possible. We next dried the DNA on a heating block for 10 min at 40 °C to let the remaining ethanol evaporate. The DNA was then resolved in 650 μl EB (10 mM Tris-HCl pH 8.5) and incubated without mixing at 4 °C overnight to resuspend into a translucent viscous gel.

### Library preparation and sequencing

Pacbio sequencing was performed by the National Genomics Infrastructure (NGI), a part of SciLifeLab in Uppsala, Sweden. In brief, 10 μg of genomic DNA was sheared into 20 kbp fragments using the Megaruptor system, followed by exo VII treatment, DNA damage repair, and end-repair before ligation with hair-pin adaptors to generate a SMRTbell™ library for circular consensus sequencing. The library was then subjected to exo treatment and PB AMPure bead wash procedures for clean-up before being size selected using the BluePippin system with a cut-off value of 9000 bp. The library was run on 11 units of a SMRTcell™ unit and sequenced on the PacBio Sequel instrument using the Sequel 2.0 polymerase and 600 min of movie time.

All our library preparations from DNA isolated using the Nanopore WGS Consortium protocol were done with an Oxford NANOPORE Rapid Sequencing Kit (SQK-RAD002, −RAD003 or -RAD004). Only cut off pipette tips were used, and great care was taken not to shear the DNA. The viscous DNA libraries were slowly pipetted onto the SpotON port and siphoned in over several minutes. The libraries were then loaded on R9.4.1 FLO-MIN106 flow cells and sequenced on a MinION Mk1B device.

### Additional sequence data

We analyzed public sequence data available at the NCBI Sequence Read Archive (SRA) database for various *Drosophila* cell line stocks and a *D. melanogaster* fly [1, 5, 32] Illumina short-read datasets, and *D. sechellia, D. simulans, D. erecta, and D.yakuba* flies [19] Nanopore long-read datasets. The NCBI SRA database accession numbers are listed in Additional file 3.

### Genome annotation and read alignment

Unless otherwise stated, *D. melanogaster* reference genome version 6.12 was used with the corresponding gene annotations [33]. Repeat annotations were obtained using RepeatMasker [34] version open-4.0.7 and the RepBase [35] database, edition 20,170,127, using the *–species fly* argument. Alignments to the reference genome were generated using Minimap2 [36] software version 2.17-r941 with the arguments *-x sr* for short-reads, *−x map-pb* for Pacbio, and *-x map-ont* for Nanopore datasets. The output was sorted using Sambamba [37] version 0.6.9 and converted to BAM format using Samtools [38] version 1.9.

### Script execution and figures

Python scripts were executed using Spyder version 3.2.4 and run interactively. Plots were generated using the matplotlib Python package [39] or Microsoft Excel version 16.0.4266.1001 unless otherwise stated. Figures were designed using Adobe Illustrator version 19.2.1.

### Copy-number comparison

Linked short-reads were aligned using Minimap2 without barcode information. Duplicates were detected from alignments using Picard [40] MarkDuplicates version 2.20.1-SNAPSHOT and with the *-BARCODE_TAG* argument to enforce awareness of barcodes. Copy-number calls were obtained from our short-read dataset using Control-FREEC [41] software version 11.4 with settings similar to those reported previously [5]. Copy-number datasets for other cell-lines were obtained from an earlier publication [5]. Since these copy-number datasets have coordinates in dmel5, coordinates were re-mapped to dmel6 using UCSC LiftOver tool [42]. To analyze copy-number agreement between samples, python script “FREEC_CN_analysis.py” was used. Copy-number calls were required to (1) be called in all three samples, (2) have > 500 bp overlap after LiftOver, (3) be < 50% annotated to repetitive sequence. Sample copy-number agreement/disagreement was visualized in a Venn-diagram using the Pyvenn (https://github.com/tctianchi/pyvenn) package. Similarly, our S2-DRSC and Sg4 and Kc167 results were compared, omitting sex-chromosomes due to the different sex karyotype.

### SNP and structural variant calling

Linked short-reads were input to Longranger [43] software version 2.2.2 to perform barcode-aware read alignment against a repeat-masked reference genome (prepared according to instructions available at the 10X Genomics homepage under “Long Ranger Installation”) and SNP calling. To study SNPs, the Python script “longranger_freec_parse.py” was used. SNP allele fractions were computed from the number of read barcodes. To study haplotype structure, SNPs were selected in regions where the copy-number call was 4 and the read barcode coverage at the SNP position was in the tetraploid range (average autosome coverage: 87.5–112.5%). Haplotype frequency was determined from SNP read support fractions; < 1 if (0–0.125], 1 if (0.125–0.375], 2 if (0.375–0.625], 3 if (0.625–0.875], > 3 if (0.875–1). SNPs with a read support fraction equal to 1 were not analyzed since they cannot be distinguished from reference-errors at the fly level.

### Visualization of read alignments and mitochondrial genome sequence insertions

To visualize reads involved in the mitochondrial genome sequence insertions, read alignment coordinates were output from the main script of our logic implementation to include information about repetitive sequence insertions. From alignment coordinates, Python script “paint_read_rsqE_path_wPyQT.py” was used to paint the read alignments. To select reads that were involved in the mitochondrial genome sequence insertion, reads were required to span the insertion by ≥15,000 bp on both sides. Additional supportive reads were identified by querying reads spanning either insertion breakpoint by ≥10,000 bp on both sides. Supportive reads were mapped to reads spanning the mitochondrion genome sequence insertion using minimap2 and argument *-x ava-ont*.

### Estimation of haplotype complexity by de novo assembly

To roughly estimate the structural complexity of haplotypes we performed de novo assembly using Wtdbg2 [44] version 2.3 with the estimated genome size set to 160 Mbp. Our combined Pacbio and Nanopore datasets were assembled by Wtdbg. Fly datasets were obtained from [19] and pooled in various ways (see Additional file 1: Fig. S6 and Additional file 4). QUAST [45] were used on the resulting assemblies to compare contiguity to S2-DRSC assembly.

### Implementation of custom logic to call rearrangements from long-reads

To classify reads as informative (used to call variants) or contained (redundant within a longer read), Minimap2 was used to perform all-versus-all read alignment of Pacbio and Nanopore reads. Reads were considered contained if fully mapped onto a longer read (queried within 750 bp from read head/tail to alleviate mapping errors at read ends). To align reads to the reference genome, Minimap2 was used with the *–r 500* and *–no-long-join* arguments to avoid deletion/insertion of sequences reported in the CIGAR string. To identify rearrangements, informative (non-contained) read alignments were imported. The alignments were sorted by score (highest score first) and distributed on the read. If a region on the read were already represented by a previous alignment, the coordinate of the current alignment was re-calculated to that of the non-represented region only. Alignments < 100 bp were omitted. Distributed alignments were then classified as (1) repetitive if > 50% mapped to a sequence annotated as repetitive in the reference genome, or (2) “anchors” if > 1 kbp length. Non-anchor or repetitive alignments were accumulated into a “junction”. An insertion was called between two anchor-alignments containing a junction sequence. To increase stringency when calling rearrangements, non-repetitive anchor-alignments were clustered into “adjacent clustered” (AC) alignments, if within 5 kbp in the reference genome, after omitting repeat-masked sequence up to 25 kbp. Rearrangements were called between alignments of ACs > 3 kbp in size. To reduce rearrangement call redundancy, calls were clustered if anchor alignments overlapped within 500 bp and have same strand at both breakpoints. Rearrangement read support was calculated by counting the number of reads (including contained reads) spanning alignment breakpoints on the read used in rearrangement call. To filter calls due to (1) rearrangements with low frequency in the population and (2) sequencing or mapping artefacts, a criterion of > 2 informative reads and > 3 read support was applied. To call local sequence duplications, the alignments of ACs were tested for overlap on the reference genome (see Additional file 1: Fig. S2A). Alignments of reads indicating a local duplication were then clustered by overlap in the reference genome. Insertions > 1 kbp in which > 90% of the sequence was annotated to one repeat element were classified as repeat elements. The four most abundant element classes were kept, and the rest were pooled into “Other”. When quantifying the total number of bases and insertion lengths of the four most abundant classes, all insertions > 1 kbp and with > 50% annotation to an element were included.

### Genome bin copy-number and coverage

Genome coverage was obtained from long-read alignments using Bedtools [46] software version 2.28.0 with the genomecov algorithm and the*–bg* argument to output results in bedgraph format. To generate high-resolution copy-number calls, the genome was divided into 50 bp bins. Bin copy-number was inferred by querying bin read coverage.

### Chromosome overview plots

To visualize coverage and rearrangements, Pygenometracks [47] software was used. Coverage was calculated as a profile of the median read alignment coverage in 1 kbp steps in 10 kbp sliding windows. Large rearrangements were identified based on a reference genome breakpoint distance > 100 kbp. The repetitive insertion density was calculated in a 100 kbp window based on repetitive insertions of > 1 kbp with > 70% of their length annotated as a repeat and > 90% of the sequence annotated as a repeat being classified to a single repeat class.

### Genome graph reconstruction from long-read variant calls

To produce a graph representation of the genome, rearrangement calls with breakpoints in the reference genome > 100 kbp apart were used to split the reference genome into segments representing nodes in the genome graph. Connections between nodes were established from the rearrangement call and an original reference connection was established if at least three reads contained both segments within a single AC (i.e., no rearrangement). The output graph was output in GFA format and visualized using Bandage [48] software version 0.8.1. To color segments by reference genome chromosome arm, the BLAST [49] function in Bandage was used with the *D. melanogaster* reference genome as the query.

### Insertion haplotype frequency and intersection with short-read transposable element insertion calls

To analyze haplotype structure, the haplotype frequency of insertions in tetraploid regions were computed. Regions were determined tetraploid by long-read coverage (87.5–112.5% of autosome average coverage) in a 3 kbp window over the insertion breakpoints. The estimated read support for one haplotype was manually determined at 44 based on the read support distribution peaks of insertions. Haplotype frequency was computed by rounding to nearest integer of the number of supportive reads divided by 44. To separate transposable element insertions occurring in the progenitor fly stock from those occurring in the cell-line, transposable element insertion calls from the S1, S2-DRSC, and S3 short-read datasets (obtained using TEFLoN [22] software version 0.4; see below) were overlapped with transposable element insertion calls from long-reads. Call overlap was determined as + − 250 bp of the long-read call. Transposable element insertions occurring in the cell-line were determined based on a requirement for long-read calls to (1) overlap the S2-DRSC short-read call, (2) not be called in S1 or S3, (3) have a read spanning the S2-DRSC short-read call breakpoint in S1 or S3.

### Gene disruptions or gains and GO-term assignment

Gene copy-number was determined by querying read coverage (from the bedgraph coverage file) in regions of “gene” entry, omitting bedgraph entries with ≥30% annotation to a repetitive sequence, and rounding to the nearest integer after division by chromosome arm average coverage. Gene disruption was determined in transcription start or stop regions, coding regions, and < 1 kbp upstream of transcription start, and was assigned if there was either a breakpoint from any rearrangement supported by at least 10 reads or a > 10 bp region of zero coverage. Gene gain was determined by querying non-disrupted genes for copy-number greater than chromosome arm basal ploidy (> 4 for autosomes and > 2 for X.). A list of gene gain/disruption status and copy-number is provided in Additional file 6. To visualize gene functional gain and disruption, lists of gained and disrupted genes were uploaded to DAVID [17] version 6.8 for functional clustering and GO-annotation. Because the list of gained genes exceeded the maximum number of inputs (3000) for DAVID, it was randomly subsampled to 3000 genes. From the DAVID output, GO-terms and *p*-values for functional clusters were extracted and submitted to REVIGO [18] for visualization.

### Comparison of mutations in various *Drosophila melanogaster* cell-lines

Public short-read datasets were downloaded [1, 5, 32] (Additional file 3). To obtain calls of TE insertions, TEFLoN [22] software was used. TEFLoN requires a pre-processed reference genome, which was set up according to the provided instructions. Read datasets of sufficient coverage (>10X) were filtered for duplicates using Fastuniq [50] software before alignment to the TEFLoN reference using BWA [51] software version 0.7.17-r1188 (using the mem algorithm and –Y argument as specified in the TEFLoN instructions). TEFLoN subsampled the short-read datasets to account for differences in sequencing depth. To allow more cell-line samples into the comparison, we also included low coverage datasets (<10X) as a separate subsample group. The subsampled (>10X and < 10X coverage groups) BAM-files were used in all subsequent analyses. We selected TE insertions called by TEFLoN that had at least 2 supportive reads. To call SNPs, subsampled alignment files were merged using Samtools [38] software and analyzed using Freebayes [52] software version 1.1.0–60-gc15b070 with the arguments *–pooled-continuous –F 0.01 –g 10,000 –C 1*. SNPs were selected that had at least 3 supportive reads reported in any sample, and which were less than 10% annotated as repetitive sequences. An SNP was considered present in a sample if the read support fraction exceeded 50% within that sample. To call local duplications, the Python script “DUP_shortread_to_phylip.py” was used. This custom script identifies local duplication signatures by querying read-pairs that (1) align between 5 kbp to 50 kbp apart, (2) have read sequences > 95% aligned as nucleotide matches, (3) are < 70% aligned to sequences annotated as repetitive, (4) read strands as expected for a duplication; If the first read in pair was forward mapped, the second read was expected to be reverse mapped and upstream, and if the first read was reverse mapped, the second read was expected to be forward mapped and downstream. Read support for the resulting duplication calls was determined based on read pairs overlapping within 3 kbp with > 80% read length in alignment for both reads and for which the read span was > 80% similar. Duplication calls were filtered out if supported by fewer than four read pairs. Copy-number calls from [5] were filtered if less than three samples had a reported copy number. For each sample, basal chromosome arm ploidy was inferred as the median of all copy-number calls between 1 and 8. Gain of copy number was determined for calls of > = 150% of basal chromosome arm ploidy. To estimate the history of cell lines, phylogeny was computed using IQ-TREE [53] software version 2.0.3 from a binary matrix of SNP sample presence/absence (see above) using the Python script “parse_freebayes.py”. We applied bootstrapping with 1000 iterations to collapse branches with < 70 support. To visualize phylogenetic trees, IToL [54] software version 6 was used. To study the similarity of cell lines based on transposable elements, local duplications, and copy-number gains, a phylogenetic approach was adopted: using the Python scripts “DUP_shortread_to_phylip.py”, “parse_teflon.py”, and “parse_copynumber.py”, the genome was divided into 1 kbp bins and binary classification was performed based on the presence/absence of the relevant mutation in each bin. Phylogeny was then computed from the resulting binary matrices by IQ-TREE (using maximum likelihood) and branches with < 70 support after 1000 bootstrapping iterations were collapsed.

## Supplementary Information

**Additional file 1: Figure S1.** Copy-number comparison between cell lines. Comparison of copy-number calls (scored in 1 kbp bins of non-repetitive regions) between the S2-DRSC line sequenced here (PS) and (A) two other S2-DRSC lines (DM & BO) from [5], and (B) an Sg4 line (male karyotype) as well as a Kc167 line (female karyotype) from [5]. The comparison between other cell lines serve as a reference point and shows how divergent cell-line stocks can be. Although the S2-DRSC are the same stock they show a copy-number agreement of 74% and thus a discrepancy of 26%. The discrepancy observed between other S2-DRSC datasets (BM and BO, showing a 82% copy-number agreement) is similar to our dataset (PS, showing 81 and 82% agreement to BM and BO, respectively) and thus confirms that our stock is S2-DRSC. **Figure S2.** Rearrangement calling logic using long-reads. (A) Description of rearrangement calling logic using long reads. (1) Reads are classified as informative or contained (redundant); Informative reads (thick black arrow) remain after filtering out contained reads that are fully mapped (thin black arrows) onto a longer read. (2) Multiple alignments to the reference genome of an informative read (shown as colored blocks with origin from the first track indicated as colored dotted lines) are interpreted as rearrangements. An insertion of sequence (black alignment block) relative to the reference genome is indicated by a black triangle. A duplication of sequence (shown as a black horizontal sparse dotted line between two hollow arrow-heads) is indicated by the overlap of alignments (overlapping regions of red, yellow, and blue blocks). Dotted black lines indicate alignments that are adjacent on the read. (3) The rearrangement haplotype frequency is estimated by counting the number of contained reads spanning the corresponding alignment breakpoint on the informative read. Using the algorithm, rearrangements were called using Pacbio and Nanopore reads. Bar plots show the abundance (X-axis) of (B) insertion sequence lengths classified by length intervals (Y-axis), (C) fraction of insertions > 1 kbp annotated as repetitive sequences, classified by fraction intervals (Y-axis), (D) rearrangement breakpoint distance relative to the reference genome for six classes (Y-axis, Transl. = Translocation), (E) rearrangement breakpoint density per Mbp and chromosome arm (Y-axis). **Figure S3.** Gain and loss of gene functions in S2-DRSC. Enriched gene functions by GO classification of genes that were (A) gained (one or more additional copies) or (B) disrupted (rearrangement breakpoint in all haplotypes). **Figure S4.** Insertions of mitochondrial genome sequence into the nuclear genome. Shown are three SVs which insert mitochondrial genome sequence. Each panel has two sections: Shown in the top is an ultra-long read onto which the mitochondrial genome sequence insertion context (including other SVs, e.g., insertions of LTR elements, shown in green) is revealed and other reads mapping onto the ultra-long read which support the event. This view is truncated to focus on the mitochondrial genome sequence. A (★) indicates that the alignment continues past clipping. Shown in the bottom is the ordered alignments to the reference genome (regions marked with color corresponding to the top section and genes shown above as blue bars) of the ultra-long read (non-truncated). Alignment scale is indicated to the left. Arrowheads denote alignment mapping to the reference forward or reverse strand. The dotted black line shows how the alignments are connected. Green triangles denote insertion of LTR elements and are sized according to the length of the inserted element. The LTR element insertions are labeled by the most abundant element class (the > 13 kbp insertions were sometimes composed of multiple segments classified to different LTR elements). Interestingly, the read sequence at the opposite side of the mitochondrial genome insertions on the 4:th chromosome (panel C, top section) is unknown: this sequence was unmapped and did not receive hits upon online BLAST at the NCBI webpage. **Figure S5.** Copy-number at translocation breakpoints. The abundance (Y-axis) of copy-number deviations above baseline (X-axis) at translocation breakpoints showed that all translocations occurred in regions with coverage above baseline (> 2 for X, > 4 for autosomes). **Figure S6.** De novo genome assembly comparison. S2-DRSC long-read datasets were assembled using Wtdbg2 (with and without removal of read repetitive sequences; see figure Legend). Fly datasets were assembled individually and as pooled datasets. The diagram shows assembly contiguity; contig length is shown on the Y-axis and the contig’s contribution to the total assembly is shown on the X-axis. Assembly contiguity of datasets comprised of phylogenetically divergent flies can be used to roughly evaluate the haplotype complexity of S2-DRSC. Assembly contiguity is expected to decrease as more datasets and more divergent datasets are pooled. Fly assemblies show high contiguity and pooled datasets show lower contiguity. S2-DRSC has low contiguity similar to that of pooled datasets regardless of the removal of repetitive regions (identified via alignment overlap to sequence annotated as repetitive sequence) from the read sequences. **Figure S7.** SNP haplotype-frequency from linked short-reads. Abundance (Y-axis) of SNPs classified to haplotype frequency (X-axis) in autosomal tetraploid regions not annotated as repetitive sequences. SNP haplotype frequencies were determined based on the fraction of reads supporting each SNP; < 1: (0–12.5%), 1: [12.5–37.5%), 2: [37.5–67.5%), 3: [67.5–87.5%), and > 3: [87.5–100%]. SNPs supported by all reads were ignored since they were probably present in the progenitor fly stock. **Figure S8.** Read support fraction of SNPs at various cutoffs. Abundance (Y-axis) of SNP supportive read fractions (X-axis). The read support cutoff value is stated in the figure titles. The Y-scale in the *N* = 1 figure is maintained in the other figures. As the read support threshold is increased (for both reads supportive of SNP and reads supportive of reference sequence), the number of SNPs decreases, which is reflective of low-frequency cellular heterogeneity. **Figure S9.** Genome bin coverage. The figure shows the long-read coverage (X-axis) distribution density (Y-axis) in 50 bp genomic bins per chromosome (colored lines). Black vertical lines denote the coverage range for copy-number assignment. Shaded regions indicate the diploid (X) and tetraploid (autosome) copy-number coverage range. **Figure S10.** Recall ratio of transposable element insertions from short-reads. Repetitive sequence insertions called from long-reads were overlapped to calls from short-read data. The figure shows the recall ratio (Y-axis) per insertion haplotype frequency and chromosome (X-axis). **Figure S11.** Presence of local duplication, transposable element insertion and gain of copy number in cell lines. Abundance of local duplications (top), transposable element insertions (middle), and copy-number gains (bottom, data from [5]) in various cell-lines. Raw data from [1, 5, 32] was used for duplication and TE insertion analysis. The datasets vary in quality (read length and read depth) and the abundance of local duplication and TE insertion cannot be directly compared. Bars labeled with asterisks indicate that a different dataset was used due to low coverage in the data from [5]. A gray shading in duplication and TE insertion abundance indicate low coverage dataset. For details see Materials and Methods. **Figure S12.** SNP phylogenetic tree zoom-in on S-cluster. Shown is a zoom-in on S-cluster in Fig. 3A. **Figure S13.** Rearrangement strandedness. Alignments in SV calls were classified as having maintained (alignments have the same strand) or changed strands (alignments have different strands). A change of strand means an inversion event occurred. The plot shows the number (Y-axis) of rearrangements with maintained or changed strand (X-axis).

**Additional file 2. **GO-terms with *p*-values for DAVID functional clusters of disrupted/gained genes.

**Additional file 3.** Public datasets used.

**Additional file 4.** Assembly QUAST statistics.

**Additional file 5.** Multiple tables containing data used to generate figures.

**Additional file 6.** Gene copy-number and disrupted genes in S2-DRSC.

## Data Availability

All of the sequence data generated in this study have been deposited in the NCBI Sequence Read Archive (SRA) database at https://www.ncbi.nlm.nih.gov/sra and is publically available under SRA accession numbers SRR15107931–39 and under BioProject accession number PRJNA745964. The NCBI SRA database accession number for all other sequence data used in this study are declared in Additional file 3. Python scripts used in this study are available upon request, and at Github: https://github.com/jaclew/S2-DRSC-scripts.

## Abbreviations

SV: Structural variant
SNP: Single nucleotide polymorphism
Pacbio: Pacific Biosciences RSII
ONT: Oxford Nanopore Technologies
bp: Base pair
kbp: Kilo-base pair
Mbp: Mega-base pair
LTR: Long terminal repeat
GO: Gene ontology
