# Supplementary material for: Transposon activity, local duplications and propagation of structural variants across haplotypes drive the evolution of the Drosophila S2 cell line
Source: BMC Genomics. 2022 Apr 7;23:276. doi: 10.1186/s12864-022-08472-1 (PMC8991648; doi:10.1186/s12864-022-08472-1)
Supplement: Supplementary file 1 — Additional file 1: Figure S1. Copy-number comparison between cell lines. Comparison of copy-number calls (scored in 1 kbp bins of non-repetitive regions) between the S2-DRSC line sequenced here (PS) and (A) two other S2-DRSC lines (DM & BO) from [5], and (B) an Sg4 line (male karyotype) as well as a Kc167 line (female karyotype) from [5]. The comparison between other cell lines serve as a reference point and shows how divergent cell-line stocks can be. Although the S2-DRSC are the same stock they show a copy-number agreement of 74% and thus a discrepancy of 26%. The discrepancy observed between other S2-DRSC datasets (BM and BO, showing a 82% copy-number agreement) is similar to our dataset (PS, showing 81 and 82% agreement to BM and BO, respectively) and thus confirms that our stock is S2-DRSC. Figure S2. Rearrangement calling logic using long-reads. (A) Description of rearrangement calling logic using long reads. (1) Reads are classified as informative or contained (redundant); Informative reads (thick black arrow) remain after filtering out contained reads that are fully mapped (thin black arrows) onto a longer read. (2) Multiple alignments to the reference genome of an informative read (shown as colored blocks with origin from the first track indicated as colored dotted lines) are interpreted as rearrangements. An insertion of sequence (black alignment block) relative to the reference genome is indicated by a black triangle. A duplication of sequence (shown as a black horizontal sparse dotted line between two hollow arrow-heads) is indicated by the overlap of alignments (overlapping regions of red, yellow, and blue blocks). Dotted black lines indicate alignments that are adjacent on the read. (3) The rearrangement haplotype frequency is estimated by counting the number of contained reads spanning the corresponding alignment breakpoint on the informative read. Using the algorithm, rearrangements were called using Pacbio and Nanopore reads. Bar plots show the abun [file 12864_2022_8472_MOESM1_ESM.pdf]

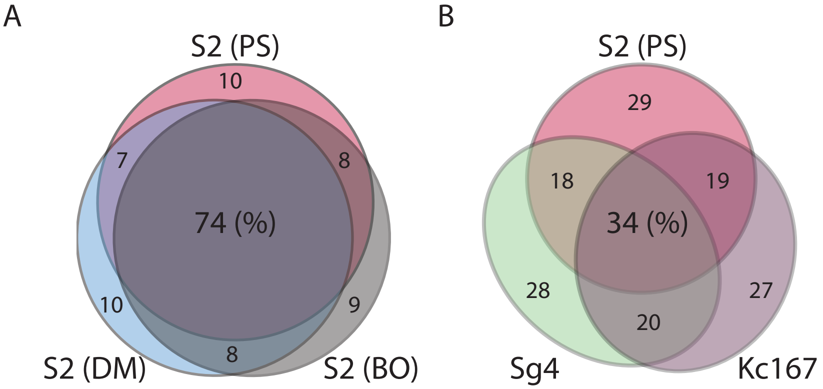
A
S2 (PS)
10
7
8
74 (%)
10
9
8
S2 (DM)
S2 (BO)
B
S2 (PS)
29
18
19
34 (%)
28
27
20
Sg4
Kc167

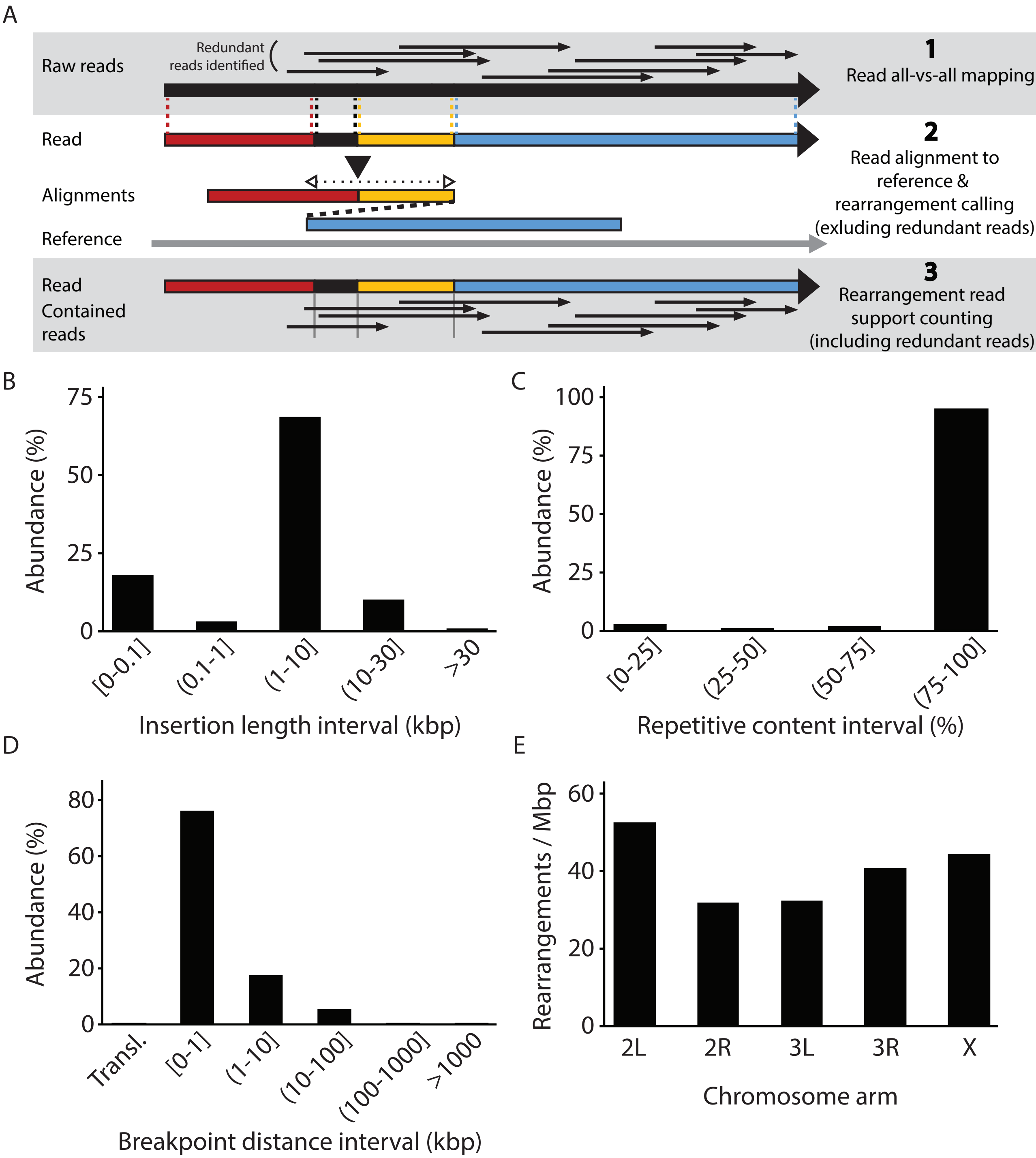
A
Raw reads
Redundant reads identified
1
Read all-vs-all mapping
Read
2
Read alignment to reference & rearrangement calling (exluding redundant reads)
Alignments
Reference
Read
Contained reads
3
Rearrangement read support counting (including redundant reads)
B
Abundance (%)
75
50
25
0
[0-0.1]
(0.1-1]
(1-10]
(10-30]
>30
Insertion length interval (kbp)
C
Abundance (%)
100
75
50
25
0
[0-25]
(25-50]
(50-75]
(75-100]
Repetitive content interval (%)
D
Abundance (%)
80
60
40
20
0
Transl.
[0-1]
(1-10]
(10-100]
(100-1000]
>1000
Breakpoint distance interval (kbp)
E
Rearrangements / Mbp
60
40
20
0
2L
2R
3L
3R
X
Chromosome arm

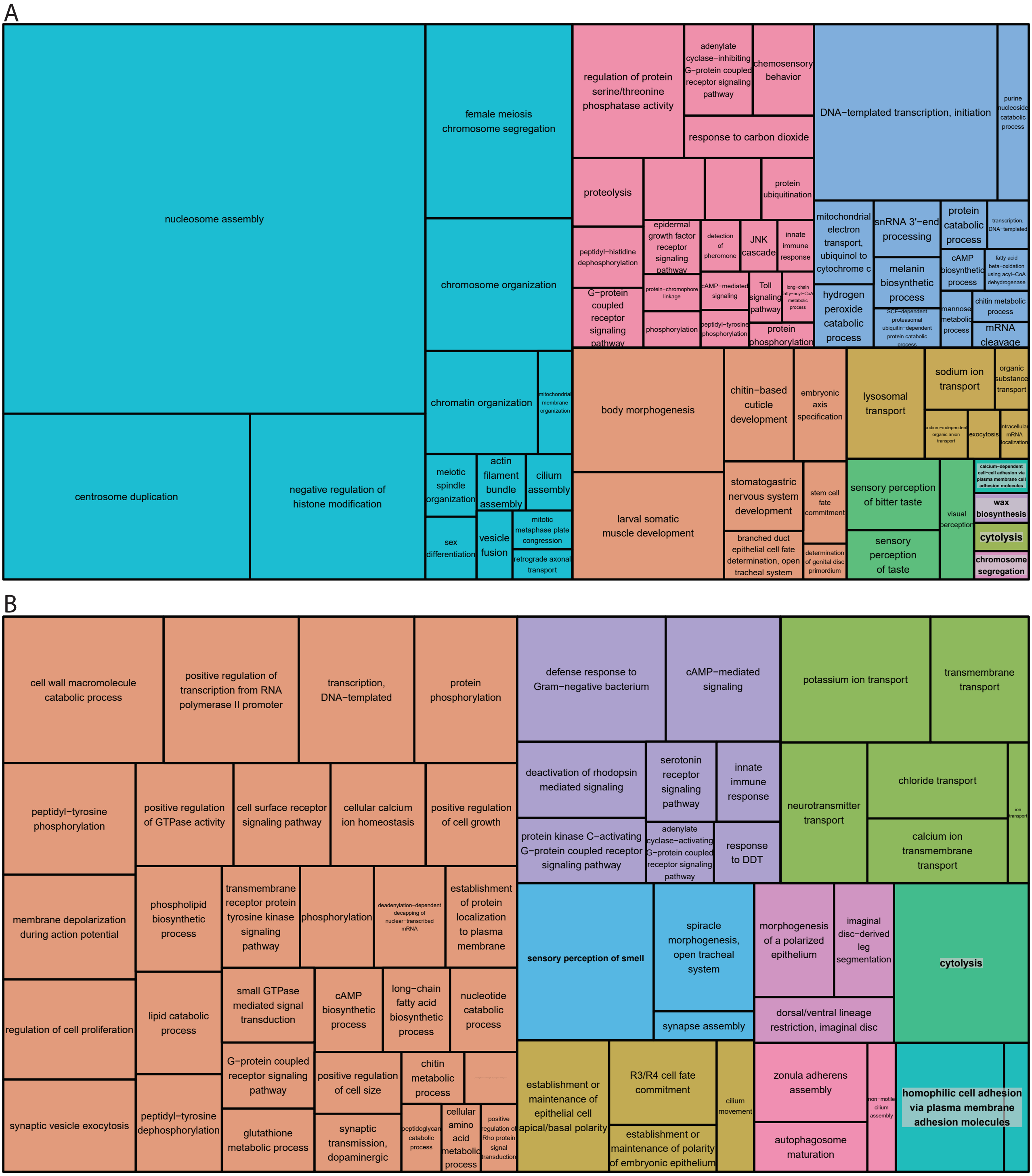

A
nucleosome assembly
female meiosis chromosome segregation
chromosome organization
chromatin organization
mitochondrial membrane organization
meiotic spindle organization
actin filament bundle assembly
cilium assembly
sex differentiation
vesicle fusion
mitotic metaphase plate congression
retrograde axonal transport
centrosome duplication
negative regulation of histone modification
regulation of protein serine/threonine phosphatase activity
adenylate cyclase−inhibiting G−protein coupled receptor signaling pathway
chemosensory behavior
response to carbon dioxide
proteolysis
protein ubiquitination
peptidyl−histidine dephosphorylation
epidermal growth factor receptor signaling pathway
detection of pheromone
JNK cascade
innate immune response
G−protein coupled receptor signaling pathway
protein−chromophore linkage
cAMP−mediated signaling
Toll signaling pathway
long−chain fatty−acyl−CoA metabolic process
phosphorylation
peptidyl−tyrosine phosphorylation
protein phosphorylation
DNA−templated transcription, initiation
purine nucleoside catabolic process
mitochondrial electron transport, ubiquinol to cytochrome c
snRNA 3'−end processing
protein catabolic process
transcription, DNA−templated
melanin biosynthetic process
cAMP biosynthetic process
fatty acid beta−oxidation using acyl−CoA dehydrogenase
hydrogen peroxide catabolic process
SCF−dependent proteasomal ubiquitin−dependent protein catabolic process
mannose metabolic process
chitin metabolic process
mRNA cleavage
body morphogenesis
chitin−based cuticle development
embryonic axis specification
lysosomal transport
sodium ion transport
organic substance transport
sodium−independent organic anion transport
exocytosis
intracellular mRNA localization
larval somatic muscle development
stomatogastric nervous system development
stem cell fate commitment
sensory perception of bitter taste
visual perception
calcium−dependent cell−cell adhesion via plasma membrane cell adhesion molecules
wax biosynthesis
cytolysis
branched duct epithelial cell fate determination, open tracheal system
determination of genital disc primordium
sensory perception of taste
chromosome segregation
B
cell wall macromolecule catabolic process
positive regulation of transcription from RNA polymerase II promoter
transcription, DNA−templated
protein phosphorylation
peptidyl−tyrosine phosphorylation
positive regulation of GTPase activity
cell surface receptor signaling pathway
cellular calcium ion homeostasis
positive regulation of cell growth
membrane depolarization during action potential
phospholipid biosynthetic process
transmembrane receptor protein tyrosine kinase signaling pathway
phosphorylation
deadenylation−dependent decapping of nuclear−transcribed mRNA
establishment of protein localization to plasma membrane
regulation of cell proliferation
lipid catabolic process
small GTPase mediated signal transduction
cAMP biosynthetic process
long−chain fatty acid biosynthetic process
nucleotide catabolic process
G−protein coupled receptor signaling pathway
positive regulation of cell size
chitin metabolic process
synaptic vesicle exocytosis
peptidyl−tyrosine dephosphorylation
glutathione metabolic process
synaptic transmission, dopaminergic
peptidoglycan catabolic process
cellular amino acid metabolic process
positive regulation of Rho protein signal transduction
defense response to Gram−negative bacterium
cAMP−mediated signaling
deactivation of rhodopsin mediated signaling
serotonin receptor signaling pathway
innate immune response
protein kinase C−activating G−protein coupled receptor signaling pathway
adenylate cyclase−activating G−protein coupled receptor signaling pathway
response to DDT
potassium ion transport
transmembrane transport
neurotransmitter transport
chloride transport
ion transport
calcium ion transmembrane transport
sensory perception of smell
spiracle morphogenesis, open tracheal system
synapse assembly
morphogenesis of a polarized epithelium
imaginal disc−derived leg segmentation
dorsal/ventral lineage restriction, imaginal disc
cytolysis
establishment or maintenance of epithelial cell apical/basal polarity
R3/R4 cell fate commitment
establishment or maintenance of polarity of embryonic epithelium
cilium movement
zonula adherens assembly
autophagosome maturation
non−motile cilium assembly
homophilic cell adhesion via plasma membrane adhesion molecules

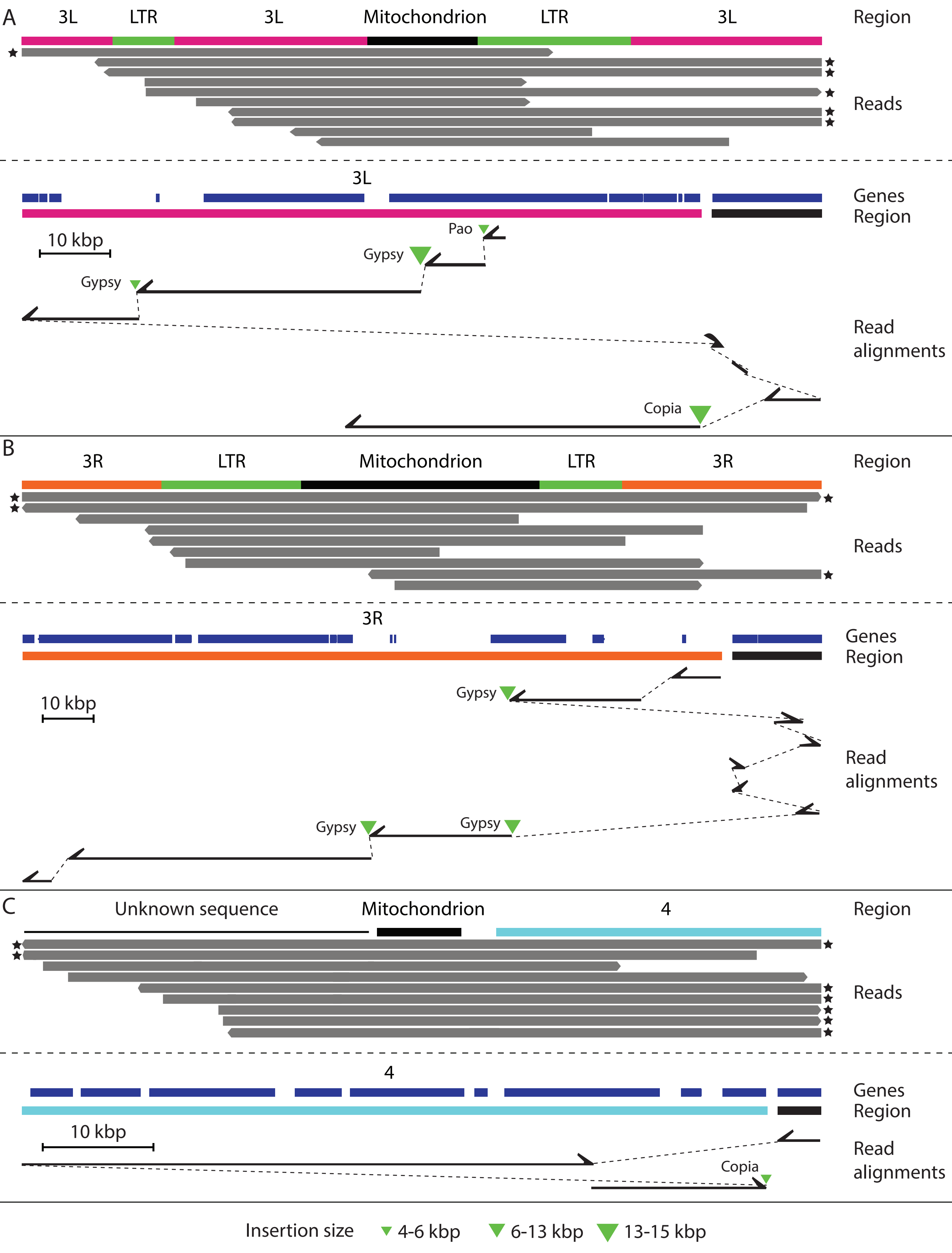
A
3L
LTR
3L
Mitochondrion
LTR
3L
Region
Reads
3L
Genes
Region
10 kbp
Pao
Gypsy
Gypsy
Read alignments
Copia
B
3R
LTR
Mitochondrion
LTR
3R
Region
Reads
3R
Genes
Region
10 kbp
Gypsy
Read alignments
Gypsy
Gypsy
C
Unknown sequence
Mitochondrion
4
Region
Reads
4
Genes
Region
10 kbp
Read alignments
Copia
Insertion size
4-6 kbp
6-13 kbp
13-15 kbp

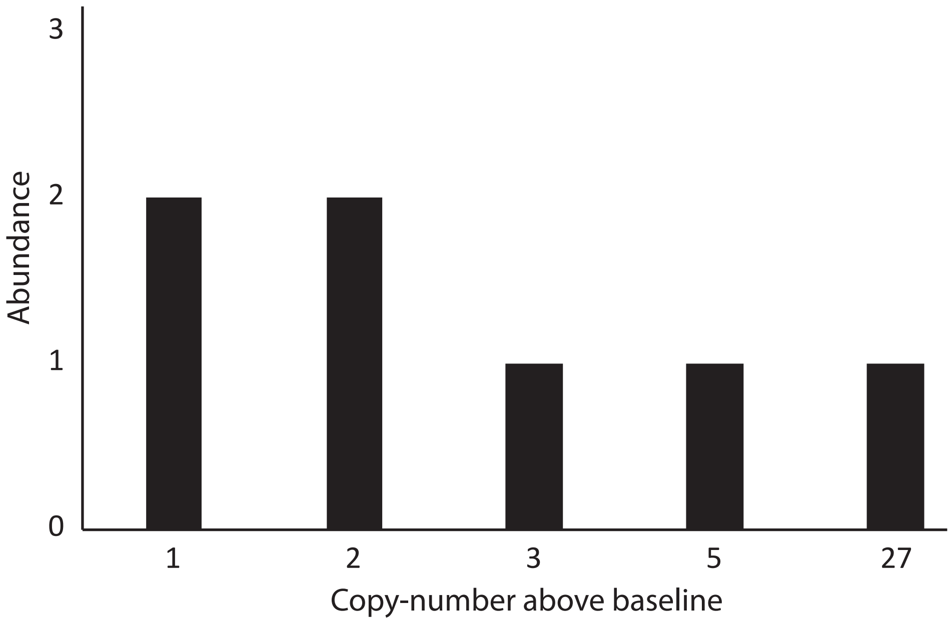
3
2
1
0
Abundance
1
2
3
5
27
Copy-number above baseline

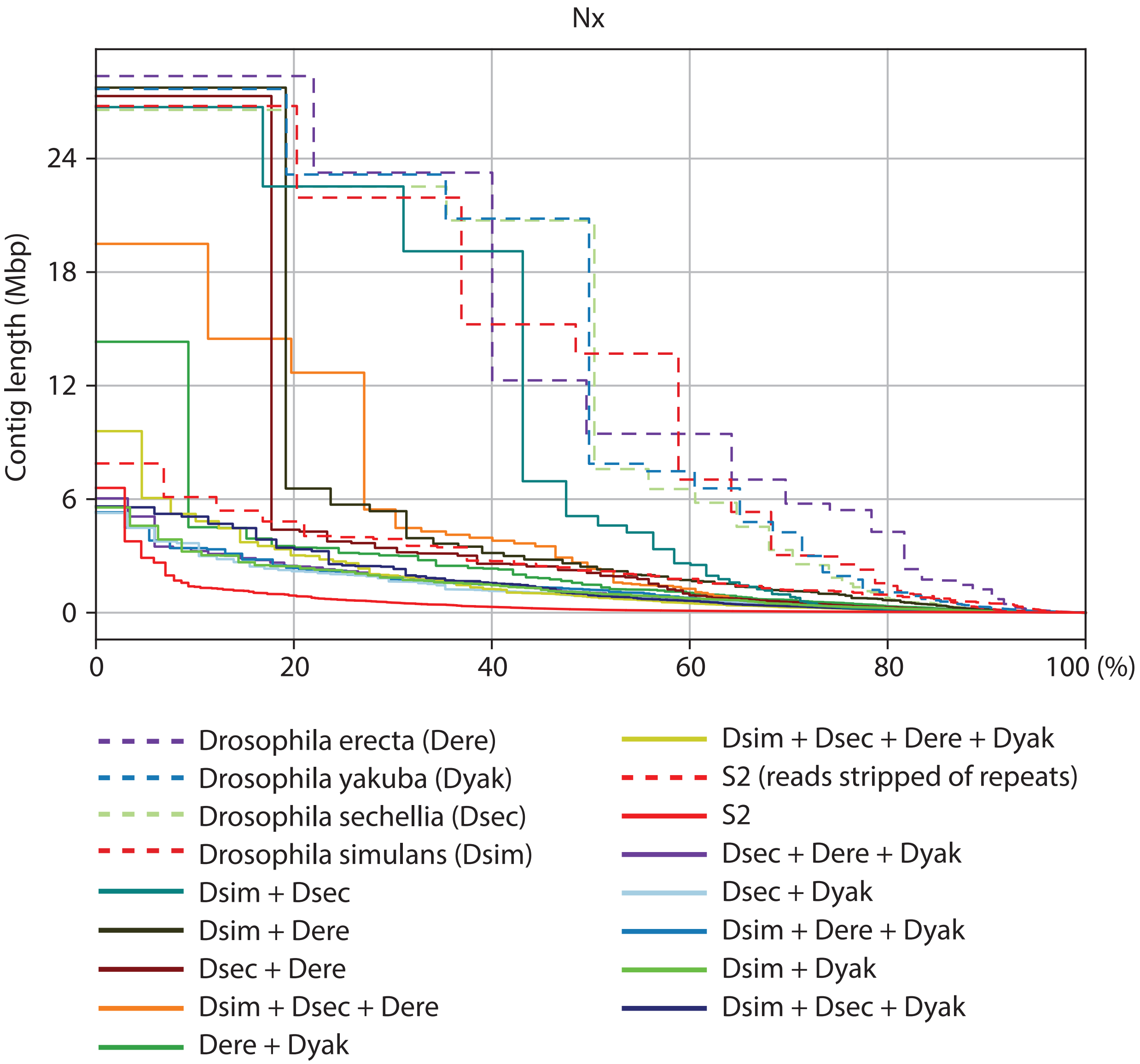

Nx
Contig length (Mbp)
24
18
12
6
0
0
20
40
60
80
100 (%)
Drosophila erecta (Dere)
Drosophila yakuba (Dyak)
Drosophila sechellia (Dsec)
Drosophila simulans (Dsim)
Dsim + Dsec
Dsim + Dere
Dsec + Dere
Dsim + Dsec + Dere
Dere + Dyak
Dsim + Dsec + Dere + Dyak
S2 (reads stripped of repeats)
S2
Dsec + Dere + Dyak
Dsec + Dyak
Dsim + Dere + Dyak
Dsim + Dyak
Dsim + Dsec + Dyak

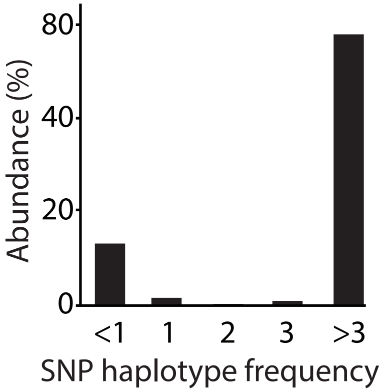

Abundance (%)
80
40
20
0
<1
1
2
3
>3
SNP haplotype frequency

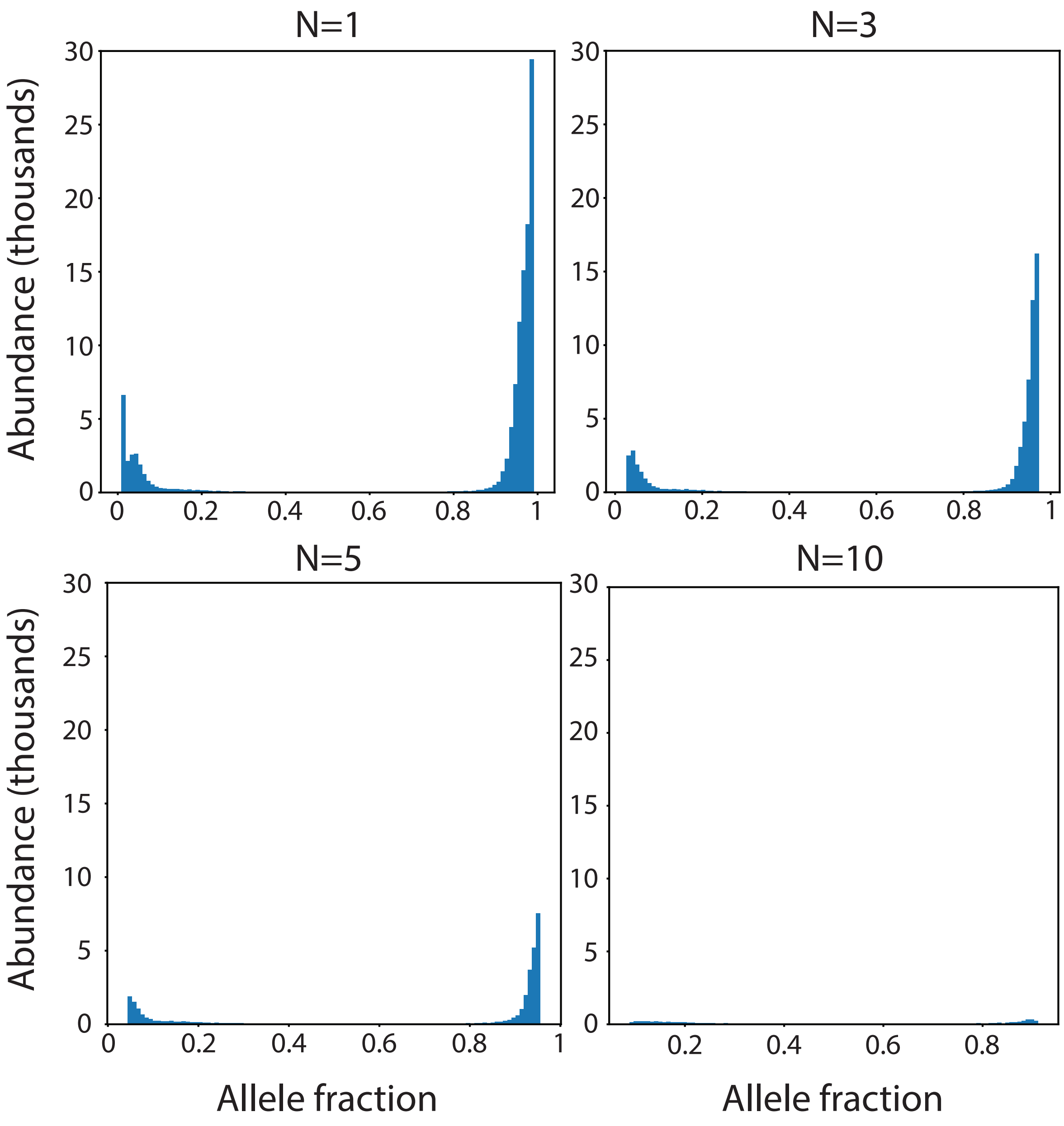
N=1
N=3
N=5
N=10
Abundance (thousands)
Abundance (thousands)
Allele fraction
Allele fraction
0
5
10
15
20
25
30
0.2
0.4
0.6
0.8
1

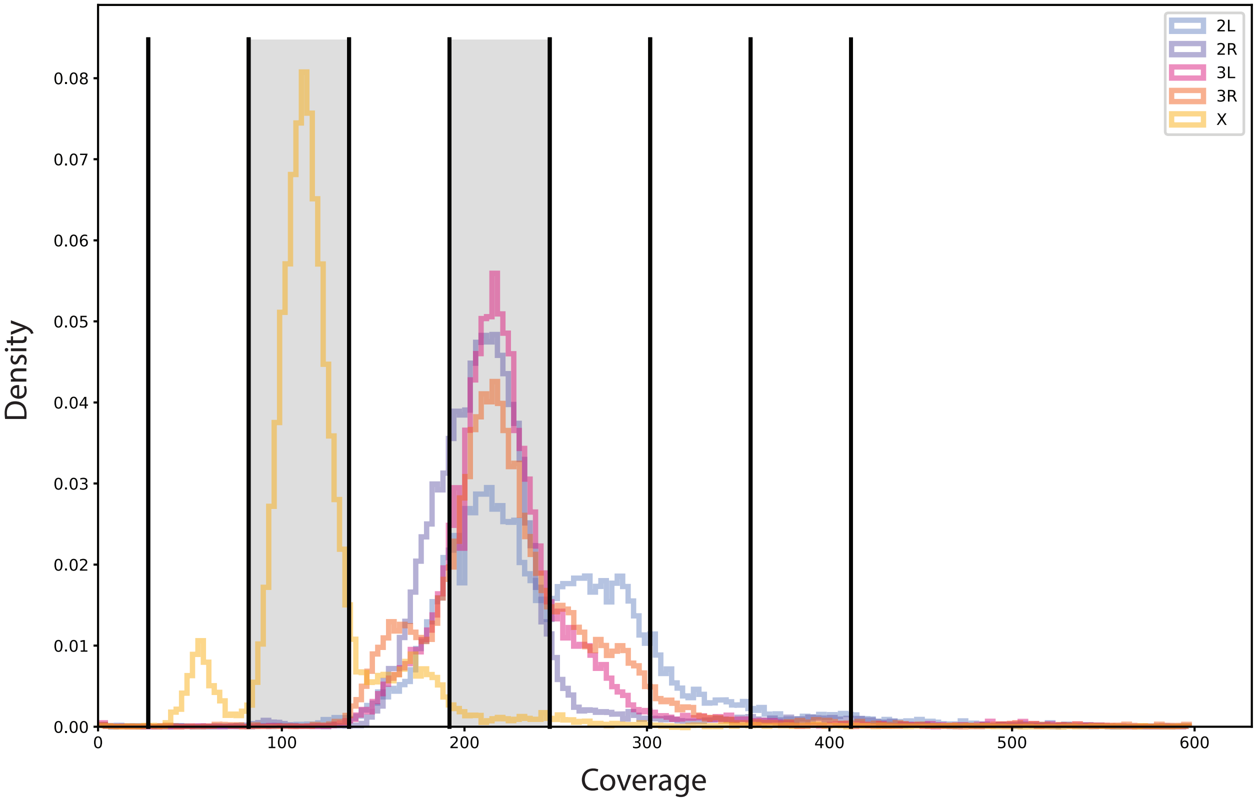

Density
Coverage
0.00
0.01
0.02
0.03
0.04
0.05
0.06
0.07
0.08
0
100
200
300
400
500
600
2L
2R
3L
3R
X

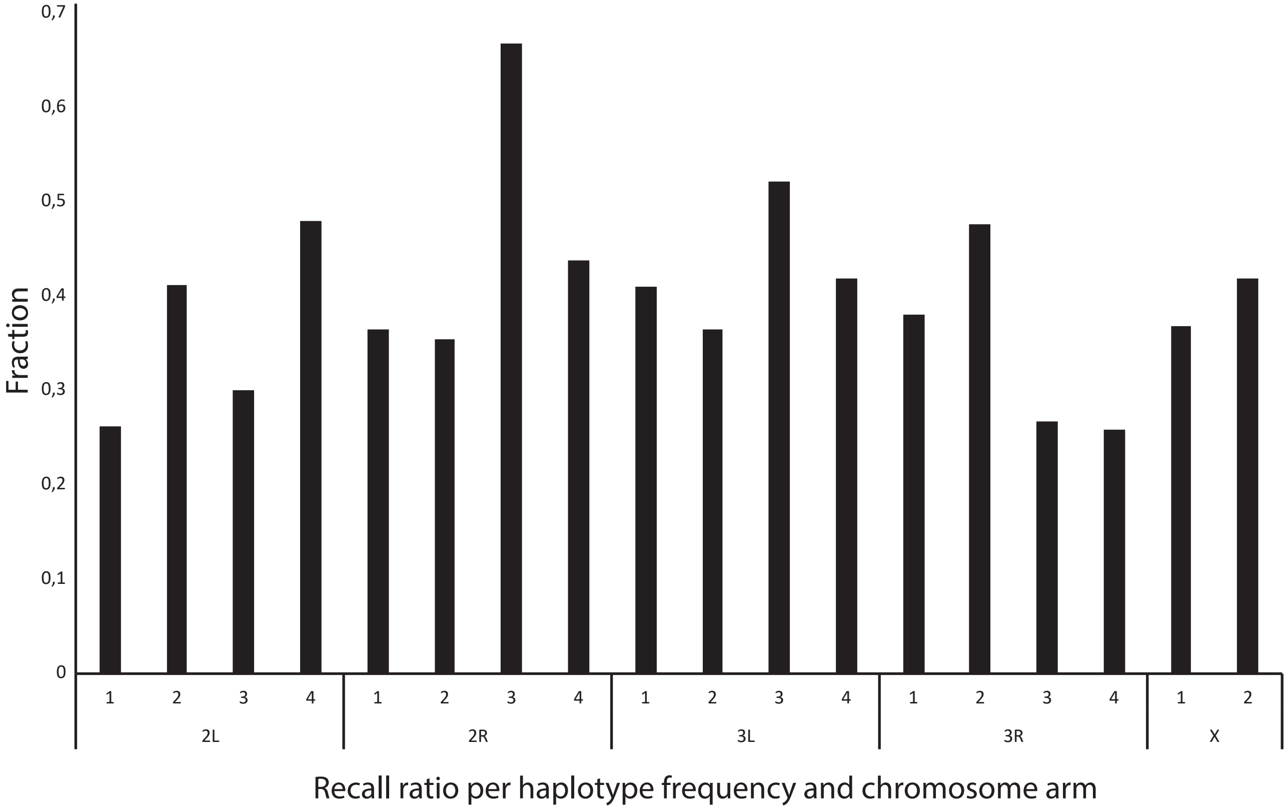

0,7
0,6
0,5
0,4
0,3
0,2
0,1
0
Fraction
1
2
3
4
2L
2R
3L
3R
X
Recall ratio per haplotype frequency and chromosome arm

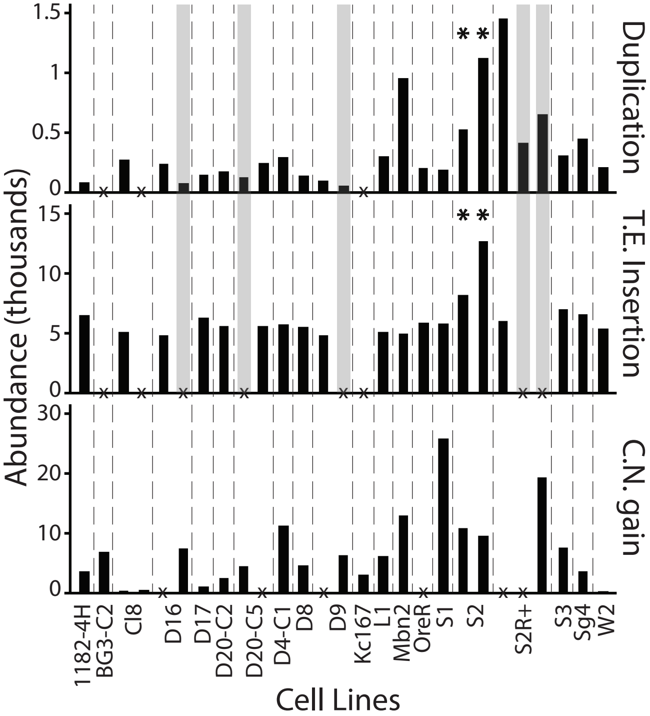
Abundance (thousands)
Duplication
T.E. Insertion
C.N. gain
1.5
1
0.5
0
15
10
5
0
30
20
10
0
1182-4H
BG3-C2
Cl8
D16
D17
D20-C2
D20-C5
D4-C1
D8
D9
Kc167
L1
Mbn2
OreR
S1
S2
S2R+
S3
Sg4
W2
Cell Lines

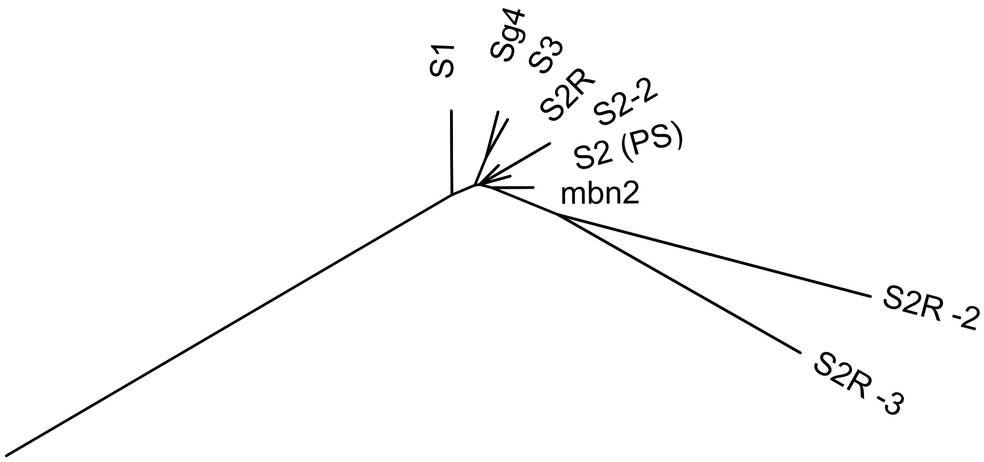
S1
Sg4
S3
S2R
S2-2
S2 (PS)
mbn2
S2R -2
S2R -3

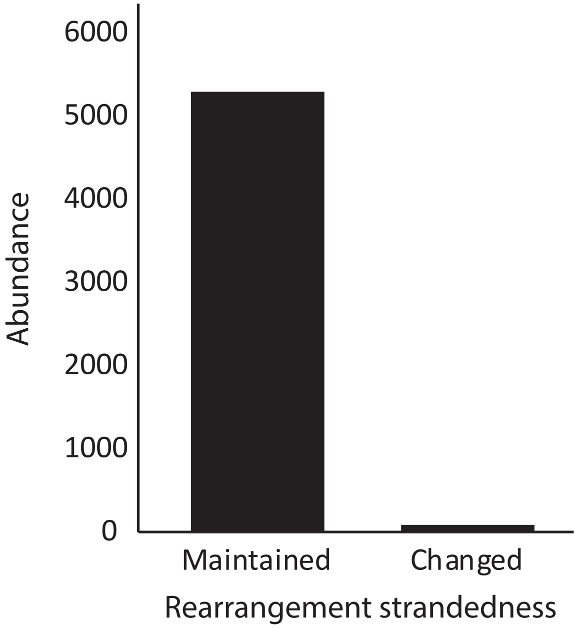

6000
5000
4000
3000
2000
1000
0
Abundance
Maintained
Changed
Rearrangement strandedness
